# Crystal structure of tricarbon­yl(μ-di­phenyl­phosphido-κ^2^ *P*:*P*)(methyl­diphenyl­silyl-κ*Si*)bis(tri­phenyl­phosphane-κ*P*)iron(II)platinum(0)(*Fe*—*Pt*)

**DOI:** 10.1107/S2056989015001565

**Published:** 2015-01-31

**Authors:** Ahmed Said Mohamed, Isabelle Jourdain, Michael Knorr, Yoann Rousselin, Marek M. Kubicki

**Affiliations:** a Institut UTINAM UMR CNRS 6213, University of Franche-Comté, 16 route de Gray, Besançon 25030, France; b ICMUB UMR CNRS 6302, University of Burgundy, 9 avenue Alain Savary, Dijon 21078, France

**Keywords:** crystal structure, heterobimetallics, phosphido bridges, iron complexes, platinum complexes, di­phenyl­methyl­silyl ligand, metal–metal bond

## Abstract

The title compound, [FePt(C_12_H_10_P)(C_13_H_13_Si)(C_18_H_15_P)_2_(CO)_3_]·0.5CH_2_Cl_2_, represents an example of a phosphido-bridged heterobimetallic silyl complex; these are inter­esting precursors for the coordination and activation of small unsaturated organic mol­ecules. The μ_2_-PPh_2_ ligand spans the iron and platinum atoms, which are connected *via* a metal–metal bond of 2.7738 (4) Å. In contrast to most other complexes of the [(OC)_3_Fe(Si*R*_3_)(μ-P*R*_2_)Pt*L*_2_] family, where the iron-bound Si*R*_3_ group is *trans*-arranged with respect to the μ_2_-PPh_2_ ligand, the SiPh_2_Me ligand is roughly collinear with the Fe–Pt vector [Si—Fe—Pt = 169.07 (3)°].

## Abstract 2

The title compound belongs to the large family of heterodinuclear phosphide-bridged complexes. The Fe—Pt bond is of 2.7738 (4) Å and there is an unprecedented arrangement of the silyl ligand in a *trans*-position with respect to the metal–metal vector in the family of phosphide-bridged iron–platinum heterobimetallics.

## Chemical context   

**Figure 1:**
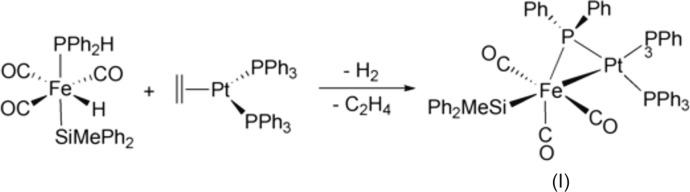
The reaction scheme for the synthesis of (I).

The bridging of metal–metal-bonded heterodinuclear complexes with μ_2_-P*R*_2_ phosphido bridges allows both the stabilization of the metal–metal bond and permits a fine-tuning of the reactivity of heterodinuclear systems by steric and electronic variation of the *R* substituents. In addition to the numerous examples of homodinuclear complexes, many μ-phosphido heterobimetallic complexes (with and without a metal–metal bond) are nowadays well documented and both their structural and reactivity features have been investigated (Stephan, 1989 ▸; He *et al.*, 1992 ▸; Comte *et al.*, 1997 ▸; Lavastre *et al.*, 1997 ▸). These compounds are usually prepared by the reaction of anionic [*L_n_M*P*R*_2_]^−^ salts with a transition metal–halide complex (Jenkins & Loeb, 1989 ▸) or by oxidative addition of the P—H bond of an [*L*_*n*_ *M*P*R*_2_H] complex across a second low-valent metal atom (Powell *et al.*, 1987 ▸). This latter route has been used to prepare the title complex [FePt(C_12_H_10_P)(C_13_H_13_Si)(C_18_H_15_P)_2_(CO)_3_]·0.5CH_2_Cl_2_ (I) and related complexes by oxidative addition of [(OC)_3_Fe(H)(Si*R*_3_)(PPh_2_H)] across [Pt(CH_2_=CH_2_)(PPh_3_)_2_] (Fig. 1 ▸). These heterodinuclear systems display an inter­esting reactivity such as ligand-induced Si*R*_3_ migration from iron to platinum, which has been studied both experimentally (Braunstein *et al.*, 1992 ▸) and theoretically (Messaoudi *et al.*, 2007 ▸). Another reactivity pattern of these electron-rich [(OC)_3_Fe(Si*R*_3_)(μ_2_-P*R*_2_)Pt(PPh_3_)_2_] compounds is their conversion to hydride-bridged μ_2_-phospido-complexes by means of protonation with HBF_4_, with concomitant cleavage of the Fe—Si*R*_3_ bond (Knorr *et al.*, 1994 ▸).
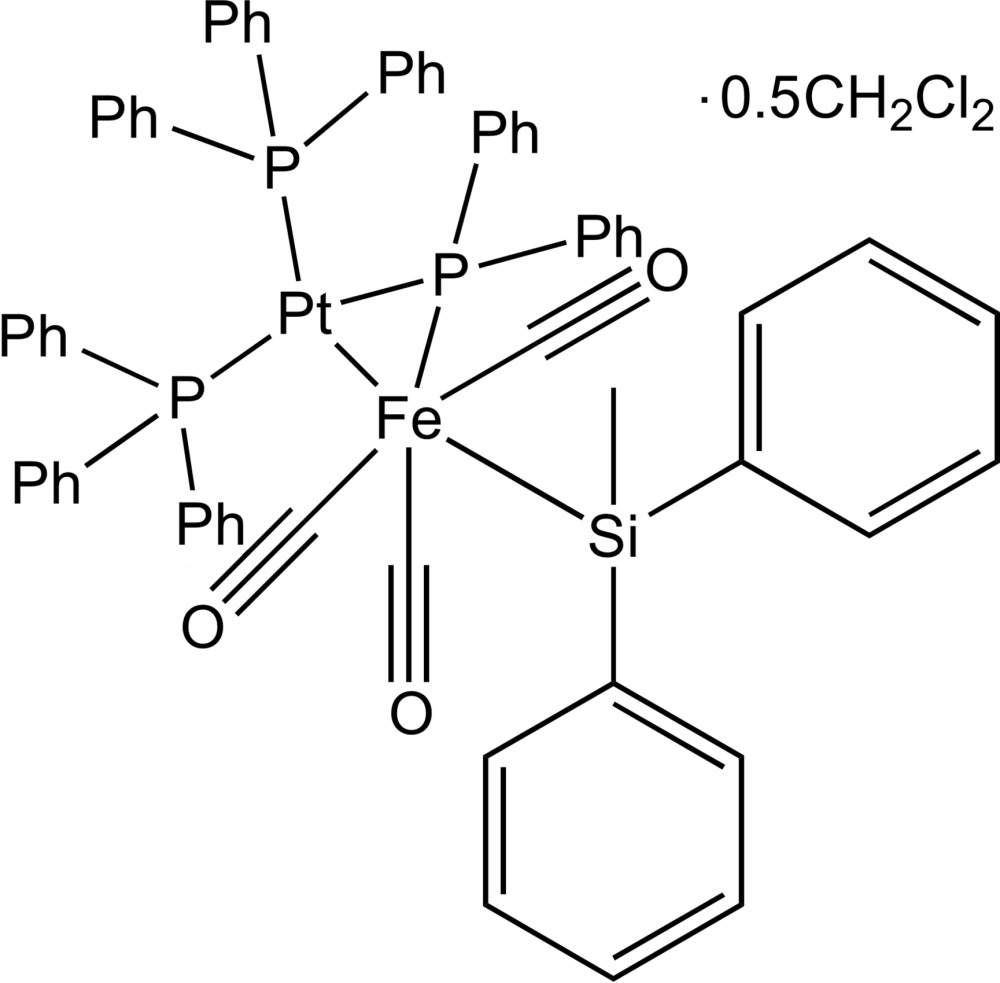


## Structural commentary   

**Figure 2:**
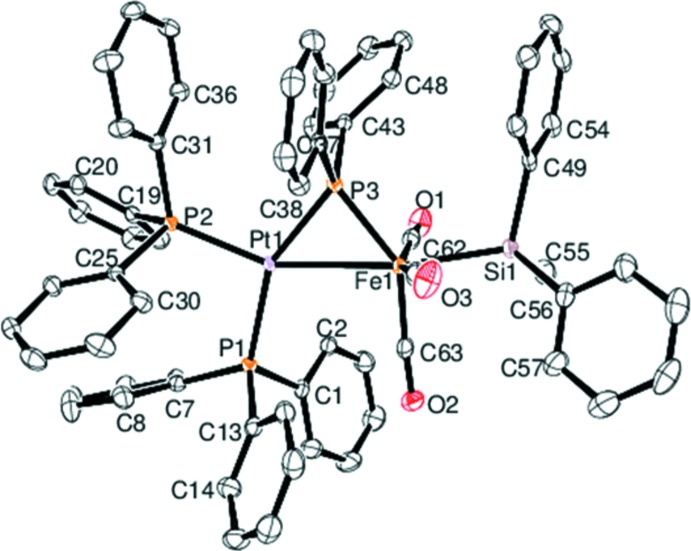
The mol­ecular structure of the title compound (I), with displacement ellipsoids shown at the 50% probabily level. H atoms have been omitted for clarity.

Compound (I) crystallized from CH_2_Cl_2_/heptane as a di­chloro­methane solvate in the triclinic space group *P* 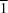. The mol­ecular structure of the organometallic mol­ecule is depicted in Fig. 2 ▸. The iron and platinum atoms are linked by a phosphide bridge and a formal metal–metal bond, whose Fe—Pt separation of 2.7738 (4) Å is somewhat longer, probably because of steric hindrance between the Ph groups of the PPh_3_ and PPh_2_ ligands, than those reported for [(OC)_3_Fe(SiPh_3_)(μ-PPh_2_)Pt(PMe_3_)_2_] [Fe—Pt = 2.701 (2) Å; Knorr *et al.*, 1994 ▸], [(OC)_3_Fe(SiPh_3_)(μ-PPh_2_)Pt{Ph_2_C(=CH_2_)PPh_2_}] [Fe—Pt = 2.659 (2) Å; Knorr *et al.*, 1994 ▸], [(OC)_3_Fe(SiPh_3_)(μ-PPh_2_)Pt(C N-Xyl­yl)(PPh_3_)] [Fe—Pt = 2.631 (1) Å; Braunstein *et al.*, 2000 ▸] and [(OC)_3_Fe(SiPh_3_)(μ-PPh_2_)Pt(CO)(PPh_3_)] [Fe—Pt = 2.620 (2) Å; Reinhard *et al.*, 1993 ▸]. The Fe—Si bond length of 2.3497 (9) Å is quite comparable with the Fe—Si bond lengths in the latter four compounds, which range from 2.330 (1) to 2.356 (3) Å. However, a striking difference concerns the relative position of the Si*R*_3_ substituent with respect to the bridging PPh_2_ group. Whereas in all four SiPh_3_-bearing complexes the silyl group is in a *trans*-position with respect to the PPh_2_ bridge, the SiPh_*2*_Me ligand of (I) is roughly colinear with the Fe–Pt vector, the Si—Fe—Pt angle being 169.07 (3)°. The P—Fe—Si angle in (I) amounts to 119.32 (3)°, whilst that of [(OC)_3_Fe(SiPh_3_)(μ-PPh_2_)Pt(C N-Xyl­yl)(PPh_3_)] [175.1 (1)°; Braunstein *et al.*, 2000 ▸] is close to a theoretical linear *trans*-arrangement.

## Supra­molecular features   

The crystal structure of (I) is built of discrete dimetallic mol­ecules without significant specific inter­molecular inter­actions.

## Database survey   

Other examples of crystallographically characterized μ-PPh_2_ Fe–Pt complexes featuring a metal–metal bond are [(OC)_3_(H)Fe(μ-PPh_2_)Pt(PPh_3_)_2_] (Powell *et al.*, 1987 ▸), [(OC)_3_Fe(SiPh_3_)(μ-PPh_2_)Pt(1,5-COD)] (COD = cyclo­octa­diene) (Braunstein *et al.*, 1995 ▸) and [NMe_4_][(OC)_3_{(MeO)_3_Si}Fe(μ-PPh_2_)Pt{Ph_2_PCH=C(O)Ph}] (Braunstein *et al.*, 1999 ▸). There is also one example of a heterodinuclear μ-PCy_2_ complex, namely [(OC)_3_(Cl)Fe(μ-PCy_2_)Pt(PEt_3_)_2_] (Jenkins *et al.*, 1990 ▸).

## Synthesis and crystallization   

The synthesis of (I) has been already published (Reinhard *et al.*, 1993 ▸). We synthesized (I) in a somewhat improved manner by reaction of [(OC)_3_Fe(H)(SiMePh_2_)(PPh_2_H)] (462 mg, 1 mmol) with [Pt(CH_2_=CH_2_)(PPh_3_)_2_] (749 mg, 1 mmol) in toluene (Fig. 1 ▸). The solution was stirred at 298 K for 1h and then concentrated until precipitation started. The precipitation of product (I) was completed by addition of hexane. The resulting yellow powder was filtered off, rinsed with hexane and dried under vacuum (969 mg, 78% yield). Suitable crystals were obtained by layering a CH_2_Cl_2_ solution with heptane and storing at 278 K in a refrigerator.

## Refinement   

Crystal data, data collection and structure refinement details are summarized in Table 1 ▸. All H atoms were placed in calculated positions and allowed to ride on their parent atoms. C—H distances were set to 0.95 Å (aromatic) and 0.98 Å (meth­yl) with *U_iso_*(H) = *xU_eq_*(C), where *x* = 1.5 for methyl and 1.2 for aromatic H atoms. The CH_2_Cl_2_ solvent mol­ecule has half occupancy and is disordered over two sites related by an inversion centre. Similar *U_ij_* constraints were applied within the disordered parts of di­chloro­methane solvent by using an EADP constraint to maintain a reasonable model.

## Supplementary Material

Crystal structure: contains datablock(s) I. DOI: 10.1107/S2056989015001565/gk2624sup1.cif

Structure factors: contains datablock(s) I. DOI: 10.1107/S2056989015001565/gk2624Isup2.hkl

CCDC reference: 1045140

Additional supporting information:  crystallographic information; 3D view; checkCIF report

## Figures and Tables

**Table 1:** Experimental details

Crystal data	
Chemical formula	[FePt(C_12_H_10_P)(C_13_H_13_Si)(C_18_H_15_P)_2_(CO)_3_]0.5CH_2_Cl_2_
*M*_r_	1284.47
Crystal system, space group	Triclinic, *P* 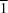
Temperature (K)	115
*a*, *b*, *c* ()	10.3522(6), 13.0010(8), 21.9803(14)
, , ()	99.823(2), 99.061(2), 102.677(2)
*V* (^3^)	2784.8(3)
*Z*	2
Radiation type	Mo *K*
(mm^1^)	2.97
Crystal size (mm)	0.15 0.05 0.02

Data collection	
Diffractometer	Nonius Kappa APEXII
Absorption correction	Multi-scan (*SADABS*; Bruker, 2008 ▸)
*T*_min_, *T*_max_	0.64, 0.74
No. of measured, independent and observed [*I* > 2(*I*)] reflections	89421, 12883, 11264
*R*_int_	0.053
(sin /)_max_ (^1^)	0.653

Refinement	
*R*[*F* ^2^ > 2(*F* ^2^)], *wR*(*F* ^2^), *S*	0.027, 0.063, 1.06
No. of reflections	12883
No. of parameters	671
H-atom treatment	H-atom parameters constrained
_max_, _min_ (e ^3^)	1.13, 1.29

Computer programs: *APEX2* and *SAINT* (Bruker, 2008 ▸), *SHELXS97* (Sheldrick, 2008 ▸), *SHELXL2014* (Sheldrick, 2015 ▸), *ORTEP-3 for Windows* (Farrugia, 2012 ▸) and *OLEX2* (Dolomanov *et al.*, 2009 ▸).

## supplementary crystallographic information

### Crystal data

**Table:**

[FePt(C_12_H_10_P)(C_13_H_13_Si)(C_18_H_15_P)_2_(CO)_3_]·0.5CH_2_Cl_2_	*Z* = 2
*M**_r_* = 1284.47	*F*(000) = 1290
Triclinic, *P*1	*D*_x_ = 1.532 Mg m^−^^3^
*a* = 10.3522 (6) Å	Mo *K*α radiation, λ = 0.71073 Å
*b* = 13.0010 (8) Å	Cell parameters from 9768 reflections
*c* = 21.9803 (14) Å	θ = 2.4–27.2°
α = 99.823 (2)°	µ = 2.97 mm^−^^1^
β = 99.061 (2)°	*T* = 115 K
γ = 102.677 (2)°	Prism, clear dark red
*V* = 2784.8 (3) Å^3^	0.15 × 0.05 × 0.02 mm

### Data collection

**Table:**

Nonius Kappa APEXII diffractometer	12883 independent reflections
Radiation source: X-ray tube, Siemens KFF Mo 2K-180	11264 reflections with *I* > 2σ(*I*)
Graphite monochromator	*R*_int_ = 0.053
Detector resolution: 9 pixels mm^-1^	θ_max_ = 27.6°, θ_min_ = 2.8°
φ and ω scans'	*h* = −13→13
Absorption correction: multi-scan (*SADABS*; Bruker, 2008)	*k* = −16→16
*T*_min_ = 0.64, *T*_max_ = 0.74	*l* = −28→28
89421 measured reflections	

### Refinement

**Table:**

Refinement on *F*^2^	3 constraints
Least-squares matrix: full	Hydrogen site location: inferred from neighbouring sites
*R*[*F*^2^ > 2σ(*F*^2^)] = 0.027	H-atom parameters constrained
*wR*(*F*^2^) = 0.063	*w* = 1/[σ^2^(*F*_o_^2^) + (0.0278*P*)^2^ + 3.2483*P*] where *P* = (*F*_o_^2^ + 2*F*_c_^2^)/3
*S* = 1.06	(Δ/σ)_max_ = 0.001
12883 reflections	Δρ_max_ = 1.13 e Å^−^^3^
671 parameters	Δρ_min_ = −1.29 e Å^−^^3^
0 restraints	

### Special details

**Table:**

Geometry. All e.s.d.'s (except the e.s.d. in the dihedral angle between two l.s. planes) are estimated using the full covariance matrix. The cell e.s.d.'s are taken into account individually in the estimation of e.s.d.'s in distances, angles and torsion angles; correlations between e.s.d.'s in cell parameters are only used when they are defined by crystal symmetry. An approximate (isotropic) treatment of cell e.s.d.'s is used for estimating e.s.d.'s involving l.s. planes.

### Fractional atomic coordinates and isotropic or equivalent isotropic displacement parameters (Å^2^)

**Table:**

	*x*	*y*	*z*	*U*_iso_*/*U*_eq_	Occ. (<1)
C1	0.1586 (3)	0.6037 (2)	0.37770 (14)	0.0155 (6)	
C2	0.0216 (3)	0.5753 (2)	0.35048 (15)	0.0178 (6)	
H2	−0.0073	0.5477	0.3063	0.021*	
C3	−0.0736 (3)	0.5873 (3)	0.38767 (16)	0.0250 (7)	
H3	−0.1670	0.5686	0.3689	0.030*	
C4	−0.0317 (4)	0.6265 (3)	0.45199 (17)	0.0307 (8)	
H4	−0.0964	0.6337	0.4776	0.037*	
C5	0.1042 (4)	0.6550 (3)	0.47889 (16)	0.0318 (8)	
H5	0.1326	0.6816	0.5231	0.038*	
C6	0.1997 (3)	0.6455 (3)	0.44221 (15)	0.0245 (7)	
H6	0.2932	0.6674	0.4611	0.029*	
C7	0.3470 (3)	0.7318 (2)	0.32479 (14)	0.0166 (6)	
C8	0.4762 (3)	0.7690 (3)	0.31449 (15)	0.0235 (7)	
H8	0.5343	0.7219	0.3110	0.028*	
C9	0.5205 (4)	0.8755 (3)	0.30925 (19)	0.0328 (9)	
H9	0.6089	0.9007	0.3019	0.039*	
C10	0.4375 (4)	0.9447 (3)	0.31459 (19)	0.0344 (9)	
H10	0.4684	1.0171	0.3105	0.041*	
C11	0.3098 (4)	0.9093 (3)	0.32577 (17)	0.0293 (8)	
H11	0.2530	0.9572	0.3300	0.035*	
C12	0.2646 (3)	0.8032 (3)	0.33081 (15)	0.0221 (7)	
H12	0.1764	0.7788	0.3385	0.027*	
C13	0.4226 (3)	0.5582 (3)	0.37477 (14)	0.0178 (6)	
C14	0.5117 (3)	0.6346 (3)	0.42404 (15)	0.0234 (7)	
H14	0.5029	0.7066	0.4322	0.028*	
C15	0.6140 (3)	0.6062 (3)	0.46162 (16)	0.0294 (8)	
H15	0.6736	0.6586	0.4957	0.035*	
C16	0.6291 (3)	0.5025 (3)	0.44955 (18)	0.0323 (9)	
H16	0.6985	0.4834	0.4756	0.039*	
C17	0.5430 (3)	0.4256 (3)	0.39946 (18)	0.0290 (8)	
H17	0.5545	0.3544	0.3906	0.035*	
C18	0.4396 (3)	0.4537 (3)	0.36219 (16)	0.0215 (7)	
H18	0.3804	0.4013	0.3280	0.026*	
C19	0.2220 (3)	0.6610 (2)	0.14442 (14)	0.0141 (6)	
C20	0.2587 (3)	0.7106 (2)	0.09621 (15)	0.0171 (6)	
H20	0.3094	0.6801	0.0689	0.020*	
C21	0.2218 (3)	0.8040 (3)	0.08772 (15)	0.0204 (7)	
H21	0.2481	0.8377	0.0549	0.025*	
C22	0.1463 (3)	0.8487 (3)	0.12713 (16)	0.0233 (7)	
H22	0.1233	0.9140	0.1222	0.028*	
C23	0.1050 (3)	0.7973 (3)	0.17353 (16)	0.0231 (7)	
H23	0.0510	0.8263	0.1996	0.028*	
C24	0.1420 (3)	0.7037 (2)	0.18217 (14)	0.0175 (6)	
H24	0.1127	0.6685	0.2139	0.021*	
C25	0.4622 (3)	0.5889 (2)	0.18633 (13)	0.0124 (6)	
C26	0.5396 (3)	0.6848 (2)	0.17855 (15)	0.0184 (6)	
H26	0.4973	0.7310	0.1580	0.022*	
C27	0.6792 (3)	0.7145 (3)	0.20055 (15)	0.0210 (7)	
H27	0.7312	0.7815	0.1958	0.025*	
C28	0.7418 (3)	0.6471 (3)	0.22915 (16)	0.0235 (7)	
H28	0.8371	0.6673	0.2438	0.028*	
C29	0.6662 (3)	0.5501 (3)	0.23649 (16)	0.0248 (7)	
H29	0.7098	0.5032	0.2558	0.030*	
C30	0.5266 (3)	0.5205 (2)	0.21581 (15)	0.0183 (6)	
H30	0.4748	0.4542	0.2216	0.022*	
C31	0.2576 (3)	0.4652 (2)	0.08079 (13)	0.0130 (6)	
C32	0.3652 (3)	0.4374 (3)	0.05658 (15)	0.0198 (6)	
H32	0.4525	0.4552	0.0829	0.024*	
C33	0.3446 (3)	0.3835 (3)	−0.00619 (15)	0.0238 (7)	
H33	0.4185	0.3659	−0.0226	0.029*	
C34	0.2182 (3)	0.3557 (2)	−0.04440 (15)	0.0209 (7)	
H34	0.2049	0.3184	−0.0869	0.025*	
C35	0.1103 (3)	0.3819 (3)	−0.02095 (15)	0.0218 (7)	
H35	0.0229	0.3627	−0.0473	0.026*	
C36	0.1304 (3)	0.4362 (3)	0.04104 (14)	0.0184 (6)	
H36	0.0560	0.4540	0.0568	0.022*	
C37	0.1517 (3)	0.2170 (2)	0.13155 (14)	0.0155 (6)	
C38	0.2858 (3)	0.2223 (3)	0.15870 (15)	0.0196 (6)	
H38	0.3257	0.2676	0.1990	0.024*	
C39	0.3610 (3)	0.1630 (3)	0.12791 (16)	0.0228 (7)	
H39	0.4516	0.1676	0.1469	0.027*	
C40	0.3032 (3)	0.0968 (3)	0.06910 (16)	0.0247 (7)	
H40	0.3538	0.0547	0.0481	0.030*	
C41	0.1728 (3)	0.0918 (3)	0.04094 (16)	0.0243 (7)	
H41	0.1343	0.0470	0.0003	0.029*	
C42	0.0966 (3)	0.1521 (3)	0.07165 (15)	0.0198 (6)	
H42	0.0071	0.1490	0.0517	0.024*	
C43	−0.0975 (3)	0.2911 (2)	0.12651 (14)	0.0148 (6)	
C44	−0.1362 (3)	0.3862 (2)	0.12109 (15)	0.0184 (6)	
H44	−0.0738	0.4540	0.1394	0.022*	
C45	−0.2639 (3)	0.3830 (3)	0.08942 (16)	0.0239 (7)	
H45	−0.2887	0.4482	0.0859	0.029*	
C46	−0.3560 (3)	0.2844 (3)	0.06278 (16)	0.0242 (7)	
H46	−0.4437	0.2822	0.0409	0.029*	
C47	−0.3202 (3)	0.1894 (3)	0.06799 (15)	0.0216 (7)	
H47	−0.3833	0.1219	0.0498	0.026*	
C48	−0.1916 (3)	0.1929 (3)	0.09989 (14)	0.0177 (6)	
H48	−0.1676	0.1274	0.1036	0.021*	
C49	−0.1895 (3)	0.0392 (2)	0.21876 (15)	0.0177 (6)	
C50	−0.1184 (3)	−0.0128 (2)	0.17901 (15)	0.0207 (7)	
H50	−0.0236	−0.0019	0.1923	0.025*	
C51	−0.1827 (4)	−0.0793 (3)	0.12126 (16)	0.0253 (7)	
H51	−0.1321	−0.1138	0.0956	0.030*	
C52	−0.3210 (4)	−0.0958 (3)	0.10066 (16)	0.0272 (8)	
H52	−0.3653	−0.1412	0.0609	0.033*	
C53	−0.3936 (3)	−0.0457 (3)	0.13849 (17)	0.0262 (7)	
H53	−0.4882	−0.0565	0.1246	0.031*	
C54	−0.3284 (3)	0.0206 (3)	0.19708 (16)	0.0227 (7)	
H54	−0.3799	0.0540	0.2228	0.027*	
C55	−0.2275 (3)	0.1690 (3)	0.34062 (17)	0.0267 (7)	
H55A	−0.2952	0.1918	0.3132	0.040*	
H55B	−0.1838	0.2277	0.3773	0.040*	
H55C	−0.2717	0.1052	0.3548	0.040*	
C56	−0.0169 (3)	0.0523 (3)	0.34648 (15)	0.0214 (7)	
C57	0.0796 (4)	0.1001 (3)	0.40177 (17)	0.0322 (8)	
H57	0.1178	0.1758	0.4106	0.039*	
C58	0.1216 (4)	0.0408 (3)	0.44428 (19)	0.0412 (10)	
H58	0.1879	0.0757	0.4813	0.049*	
C59	0.0668 (4)	−0.0689 (3)	0.43261 (19)	0.0374 (9)	
H59	0.0942	−0.1099	0.4619	0.045*	
C60	−0.0282 (4)	−0.1191 (3)	0.3781 (2)	0.0363 (9)	
H60	−0.0659	−0.1949	0.3697	0.044*	
C61	−0.0684 (4)	−0.0592 (3)	0.33573 (17)	0.0274 (7)	
H61	−0.1330	−0.0951	0.2982	0.033*	
C62	−0.1061 (3)	0.3215 (2)	0.26394 (15)	0.0188 (6)	
C63	0.1099 (3)	0.3508 (2)	0.35684 (15)	0.0190 (6)	
C64	0.1618 (3)	0.1991 (3)	0.27758 (15)	0.0196 (6)	
O1	−0.2091 (2)	0.3414 (2)	0.25651 (12)	0.0302 (6)	
O2	0.1469 (3)	0.38590 (19)	0.41022 (11)	0.0283 (5)	
O3	0.2311 (3)	0.1409 (2)	0.27948 (13)	0.0334 (6)	
Si1	−0.09598 (8)	0.13518 (7)	0.29544 (4)	0.01607 (17)	
P1	0.28062 (7)	0.58904 (6)	0.32670 (4)	0.01326 (15)	
P2	0.27801 (7)	0.54250 (6)	0.16115 (3)	0.01088 (14)	
P3	0.06216 (7)	0.29705 (6)	0.17794 (4)	0.01213 (14)	
Fe1	0.05124 (4)	0.28585 (3)	0.27603 (2)	0.01319 (9)	
Pt1	0.18625 (2)	0.45986 (2)	0.23346 (2)	0.01069 (4)	
Cl1	0.4990 (4)	0.8723 (3)	0.5382 (2)	0.1272 (12)	0.5
Cl2	0.5633 (5)	1.0759 (3)	0.4954 (2)	0.1272 (12)	0.5
C65	0.4441 (7)	0.9859 (6)	0.5229 (7)	0.1272 (12)	0.5
H65A	0.3586	0.9616	0.4910	0.153*	0.5
H65B	0.4251	1.0245	0.5620	0.153*	0.5

### Atomic displacement parameters (Å^2^)

**Table:**

	*U*^11^	*U*^22^	*U*^33^	*U*^12^	*U*^13^	*U*^23^
C1	0.0187 (15)	0.0137 (14)	0.0161 (15)	0.0036 (12)	0.0080 (12)	0.0046 (12)
C2	0.0209 (15)	0.0157 (15)	0.0175 (15)	0.0036 (12)	0.0057 (12)	0.0049 (12)
C3	0.0195 (16)	0.0279 (18)	0.0266 (18)	0.0046 (14)	0.0055 (14)	0.0045 (15)
C4	0.0296 (19)	0.039 (2)	0.0279 (19)	0.0094 (16)	0.0179 (16)	0.0061 (16)
C5	0.035 (2)	0.044 (2)	0.0156 (17)	0.0088 (17)	0.0092 (15)	0.0015 (16)
C6	0.0209 (16)	0.0294 (18)	0.0202 (17)	0.0024 (14)	0.0037 (13)	0.0025 (14)
C7	0.0201 (15)	0.0143 (14)	0.0123 (14)	0.0005 (12)	0.0022 (12)	0.0007 (12)
C8	0.0257 (17)	0.0190 (16)	0.0227 (17)	0.0024 (13)	0.0084 (14)	−0.0032 (13)
C9	0.034 (2)	0.0201 (17)	0.046 (2)	0.0012 (15)	0.0241 (18)	0.0046 (16)
C10	0.044 (2)	0.0149 (17)	0.044 (2)	0.0004 (15)	0.0202 (19)	0.0047 (16)
C11	0.040 (2)	0.0166 (16)	0.035 (2)	0.0086 (15)	0.0153 (17)	0.0046 (15)
C12	0.0245 (17)	0.0171 (16)	0.0241 (17)	0.0023 (13)	0.0067 (14)	0.0045 (13)
C13	0.0148 (14)	0.0232 (16)	0.0160 (15)	0.0029 (12)	0.0047 (12)	0.0067 (13)
C14	0.0203 (16)	0.0260 (17)	0.0215 (17)	0.0045 (14)	0.0030 (13)	0.0018 (14)
C15	0.0187 (16)	0.044 (2)	0.0217 (18)	0.0036 (15)	−0.0013 (14)	0.0068 (16)
C16	0.0186 (17)	0.047 (2)	0.036 (2)	0.0093 (16)	0.0037 (15)	0.0240 (18)
C17	0.0214 (17)	0.0305 (19)	0.040 (2)	0.0076 (15)	0.0076 (16)	0.0183 (17)
C18	0.0178 (15)	0.0222 (17)	0.0247 (17)	0.0025 (13)	0.0033 (13)	0.0100 (14)
C19	0.0118 (13)	0.0122 (14)	0.0170 (15)	0.0016 (11)	0.0000 (11)	0.0041 (12)
C20	0.0119 (14)	0.0189 (15)	0.0208 (16)	0.0031 (12)	0.0029 (12)	0.0067 (13)
C21	0.0171 (15)	0.0209 (16)	0.0243 (17)	0.0028 (13)	0.0017 (13)	0.0121 (13)
C22	0.0260 (17)	0.0161 (16)	0.0292 (18)	0.0086 (13)	0.0006 (14)	0.0091 (14)
C23	0.0270 (17)	0.0193 (16)	0.0264 (18)	0.0119 (14)	0.0070 (14)	0.0048 (14)
C24	0.0187 (15)	0.0181 (15)	0.0164 (15)	0.0058 (12)	0.0028 (12)	0.0049 (12)
C25	0.0111 (13)	0.0148 (14)	0.0107 (14)	0.0033 (11)	0.0016 (11)	0.0015 (11)
C26	0.0169 (15)	0.0189 (16)	0.0193 (16)	0.0031 (12)	0.0016 (12)	0.0078 (13)
C27	0.0147 (15)	0.0238 (17)	0.0210 (17)	−0.0021 (13)	0.0027 (13)	0.0052 (13)
C28	0.0130 (15)	0.0269 (18)	0.0262 (18)	0.0031 (13)	−0.0003 (13)	0.0002 (14)
C29	0.0201 (16)	0.0218 (17)	0.0303 (19)	0.0095 (13)	−0.0034 (14)	0.0025 (14)
C30	0.0185 (15)	0.0142 (15)	0.0207 (16)	0.0040 (12)	0.0005 (13)	0.0035 (12)
C31	0.0179 (14)	0.0099 (13)	0.0114 (14)	0.0031 (11)	0.0022 (11)	0.0039 (11)
C32	0.0169 (15)	0.0258 (17)	0.0163 (16)	0.0087 (13)	−0.0001 (12)	0.0027 (13)
C33	0.0271 (17)	0.0270 (18)	0.0195 (17)	0.0138 (14)	0.0069 (14)	0.0000 (14)
C34	0.0317 (18)	0.0159 (15)	0.0139 (15)	0.0060 (13)	0.0033 (13)	0.0008 (12)
C35	0.0187 (16)	0.0228 (17)	0.0177 (16)	−0.0009 (13)	−0.0032 (13)	0.0020 (13)
C36	0.0148 (14)	0.0218 (16)	0.0172 (15)	0.0019 (12)	0.0036 (12)	0.0031 (13)
C37	0.0176 (15)	0.0109 (14)	0.0185 (15)	0.0025 (11)	0.0057 (12)	0.0039 (12)
C38	0.0184 (15)	0.0202 (16)	0.0188 (16)	0.0027 (13)	0.0035 (13)	0.0034 (13)
C39	0.0187 (16)	0.0225 (17)	0.0311 (19)	0.0078 (13)	0.0088 (14)	0.0098 (14)
C40	0.0297 (18)	0.0199 (16)	0.0305 (19)	0.0104 (14)	0.0174 (15)	0.0056 (14)
C41	0.0313 (18)	0.0171 (16)	0.0219 (17)	0.0023 (14)	0.0103 (14)	−0.0026 (13)
C42	0.0174 (15)	0.0198 (16)	0.0205 (16)	0.0026 (12)	0.0037 (13)	0.0028 (13)
C43	0.0143 (14)	0.0155 (14)	0.0147 (15)	0.0014 (11)	0.0043 (12)	0.0056 (12)
C44	0.0174 (15)	0.0152 (15)	0.0237 (17)	0.0027 (12)	0.0067 (13)	0.0062 (13)
C45	0.0214 (16)	0.0238 (17)	0.0331 (19)	0.0122 (14)	0.0085 (14)	0.0128 (15)
C46	0.0154 (15)	0.0343 (19)	0.0262 (18)	0.0087 (14)	0.0021 (13)	0.0139 (15)
C47	0.0161 (15)	0.0241 (17)	0.0211 (17)	−0.0010 (13)	0.0019 (13)	0.0048 (13)
C48	0.0176 (15)	0.0190 (15)	0.0175 (15)	0.0045 (12)	0.0045 (12)	0.0057 (12)
C49	0.0191 (15)	0.0109 (14)	0.0226 (16)	0.0000 (12)	0.0050 (13)	0.0064 (12)
C50	0.0255 (17)	0.0138 (15)	0.0237 (17)	0.0043 (13)	0.0061 (14)	0.0062 (13)
C51	0.037 (2)	0.0150 (16)	0.0245 (18)	0.0070 (14)	0.0067 (15)	0.0048 (13)
C52	0.039 (2)	0.0135 (15)	0.0216 (17)	−0.0004 (14)	−0.0046 (15)	0.0027 (13)
C53	0.0227 (17)	0.0192 (17)	0.0321 (19)	−0.0035 (13)	−0.0016 (14)	0.0112 (15)
C54	0.0211 (16)	0.0185 (16)	0.0282 (18)	0.0019 (13)	0.0051 (14)	0.0080 (14)
C55	0.0261 (18)	0.0254 (18)	0.0284 (19)	0.0028 (14)	0.0136 (15)	0.0025 (15)
C56	0.0253 (17)	0.0191 (16)	0.0231 (17)	0.0052 (13)	0.0102 (14)	0.0091 (13)
C57	0.046 (2)	0.0251 (19)	0.0237 (19)	0.0060 (16)	0.0019 (16)	0.0095 (15)
C58	0.050 (3)	0.045 (2)	0.027 (2)	0.012 (2)	−0.0002 (18)	0.0129 (18)
C59	0.050 (2)	0.040 (2)	0.039 (2)	0.024 (2)	0.0167 (19)	0.0267 (19)
C60	0.046 (2)	0.0249 (19)	0.047 (2)	0.0133 (17)	0.018 (2)	0.0179 (18)
C61	0.0331 (19)	0.0206 (17)	0.0300 (19)	0.0062 (15)	0.0092 (15)	0.0079 (15)
C62	0.0239 (17)	0.0151 (15)	0.0198 (16)	0.0057 (13)	0.0073 (13)	0.0062 (12)
C63	0.0251 (16)	0.0131 (15)	0.0190 (17)	0.0025 (12)	0.0057 (13)	0.0059 (13)
C64	0.0197 (16)	0.0199 (16)	0.0208 (16)	0.0022 (13)	0.0072 (13)	0.0097 (13)
O1	0.0258 (13)	0.0354 (14)	0.0414 (15)	0.0174 (11)	0.0157 (11)	0.0191 (12)
O2	0.0410 (14)	0.0226 (12)	0.0169 (12)	0.0016 (11)	0.0022 (11)	0.0039 (10)
O3	0.0351 (14)	0.0374 (15)	0.0449 (16)	0.0244 (12)	0.0188 (12)	0.0256 (13)
Si1	0.0184 (4)	0.0127 (4)	0.0164 (4)	0.0012 (3)	0.0053 (3)	0.0035 (3)
P1	0.0136 (4)	0.0120 (4)	0.0127 (4)	0.0010 (3)	0.0027 (3)	0.0016 (3)
P2	0.0104 (3)	0.0100 (3)	0.0117 (3)	0.0017 (3)	0.0018 (3)	0.0023 (3)
P3	0.0118 (3)	0.0101 (3)	0.0139 (4)	0.0019 (3)	0.0020 (3)	0.0025 (3)
Fe1	0.0145 (2)	0.0110 (2)	0.0140 (2)	0.00205 (16)	0.00347 (16)	0.00358 (16)
Pt1	0.01105 (5)	0.00869 (5)	0.01160 (6)	0.00084 (4)	0.00250 (4)	0.00217 (4)
Cl1	0.107 (2)	0.101 (3)	0.124 (3)	0.0166 (17)	−0.0281 (18)	−0.050 (2)
Cl2	0.107 (2)	0.101 (3)	0.124 (3)	0.0166 (17)	−0.0281 (18)	−0.050 (2)
C65	0.107 (2)	0.101 (3)	0.124 (3)	0.0166 (17)	−0.0281 (18)	−0.050 (2)

### Geometric parameters (Å, º)

**Table:**

C1—C2	1.391 (4)	C35—C36	1.384 (4)
C1—C6	1.390 (4)	C36—H36	0.9500
C1—P1	1.835 (3)	C37—C38	1.404 (4)
C2—H2	0.9500	C37—C42	1.394 (4)
C2—C3	1.393 (4)	C37—P3	1.828 (3)
C3—H3	0.9500	C38—H38	0.9500
C3—C4	1.382 (5)	C38—C39	1.381 (4)
C4—H4	0.9500	C39—H39	0.9500
C4—C5	1.379 (5)	C39—C40	1.386 (5)
C5—H5	0.9500	C40—H40	0.9500
C5—C6	1.384 (5)	C40—C41	1.376 (5)
C6—H6	0.9500	C41—H41	0.9500
C7—C8	1.387 (4)	C41—C42	1.397 (4)
C7—C12	1.396 (4)	C42—H42	0.9500
C7—P1	1.842 (3)	C43—C44	1.400 (4)
C8—H8	0.9500	C43—C48	1.393 (4)
C8—C9	1.390 (5)	C43—P3	1.827 (3)
C9—H9	0.9500	C44—H44	0.9500
C9—C10	1.378 (5)	C44—C45	1.382 (4)
C10—H10	0.9500	C45—H45	0.9500
C10—C11	1.376 (5)	C45—C46	1.387 (5)
C11—H11	0.9500	C46—H46	0.9500
C11—C12	1.384 (5)	C46—C47	1.383 (5)
C12—H12	0.9500	C47—H47	0.9500
C13—C14	1.388 (4)	C47—C48	1.391 (4)
C13—C18	1.394 (4)	C48—H48	0.9500
C13—P1	1.831 (3)	C49—C50	1.408 (4)
C14—H14	0.9500	C49—C54	1.396 (4)
C14—C15	1.393 (5)	C49—Si1	1.890 (3)
C15—H15	0.9500	C50—H50	0.9500
C15—C16	1.377 (5)	C50—C51	1.381 (5)
C16—H16	0.9500	C51—H51	0.9500
C16—C17	1.389 (5)	C51—C52	1.389 (5)
C17—H17	0.9500	C52—H52	0.9500
C17—C18	1.396 (5)	C52—C53	1.381 (5)
C18—H18	0.9500	C53—H53	0.9500
C19—C20	1.391 (4)	C53—C54	1.396 (5)
C19—C24	1.394 (4)	C54—H54	0.9500
C19—P2	1.834 (3)	C55—H55A	0.9800
C20—H20	0.9500	C55—H55B	0.9800
C20—C21	1.383 (4)	C55—H55C	0.9800
C21—H21	0.9500	C55—Si1	1.890 (3)
C21—C22	1.393 (5)	C56—C57	1.396 (5)
C22—H22	0.9500	C56—C61	1.395 (5)
C22—C23	1.383 (5)	C56—Si1	1.897 (3)
C23—H23	0.9500	C57—H57	0.9500
C23—C24	1.388 (4)	C57—C58	1.386 (5)
C24—H24	0.9500	C58—H58	0.9500
C25—C26	1.379 (4)	C58—C59	1.377 (6)
C25—C30	1.405 (4)	C59—H59	0.9500
C25—P2	1.832 (3)	C59—C60	1.381 (6)
C26—H26	0.9500	C60—H60	0.9500
C26—C27	1.394 (4)	C60—C61	1.384 (5)
C27—H27	0.9500	C61—H61	0.9500
C27—C28	1.376 (5)	C62—O1	1.145 (4)
C28—H28	0.9500	C62—Fe1	1.782 (3)
C28—C29	1.380 (5)	C63—O2	1.153 (4)
C29—H29	0.9500	C63—Fe1	1.778 (3)
C29—C30	1.390 (4)	C64—O3	1.152 (4)
C30—H30	0.9500	C64—Fe1	1.776 (3)
C31—C32	1.397 (4)	Si1—Fe1	2.3497 (9)
C31—C36	1.395 (4)	P1—Pt1	2.3346 (8)
C31—P2	1.832 (3)	P2—Pt1	2.2787 (7)
C32—H32	0.9500	P3—Fe1	2.2045 (9)
C32—C33	1.398 (4)	P3—Pt1	2.2475 (7)
C33—H33	0.9500	Fe1—Pt1	2.7738 (4)
C33—C34	1.375 (5)	Cl1—C65	1.7602
C34—H34	0.9500	Cl2—C65	1.7600
C34—C35	1.385 (4)	C65—H65A	0.9900
C35—H35	0.9500	C65—H65B	0.9900
			
C2—C1—P1	118.8 (2)	C40—C41—H41	119.8
C6—C1—C2	119.4 (3)	C40—C41—C42	120.4 (3)
C6—C1—P1	121.8 (2)	C42—C41—H41	119.8
C1—C2—H2	119.9	C37—C42—C41	120.1 (3)
C1—C2—C3	120.3 (3)	C37—C42—H42	120.0
C3—C2—H2	119.9	C41—C42—H42	120.0
C2—C3—H3	120.1	C44—C43—P3	120.0 (2)
C4—C3—C2	119.9 (3)	C48—C43—C44	118.3 (3)
C4—C3—H3	120.1	C48—C43—P3	121.0 (2)
C3—C4—H4	120.1	C43—C44—H44	119.6
C5—C4—C3	119.8 (3)	C45—C44—C43	120.9 (3)
C5—C4—H4	120.1	C45—C44—H44	119.6
C4—C5—H5	119.6	C44—C45—H45	120.0
C4—C5—C6	120.8 (3)	C44—C45—C46	120.0 (3)
C6—C5—H5	119.6	C46—C45—H45	120.0
C1—C6—H6	120.1	C45—C46—H46	120.0
C5—C6—C1	119.9 (3)	C47—C46—C45	120.1 (3)
C5—C6—H6	120.1	C47—C46—H46	120.0
C8—C7—C12	118.9 (3)	C46—C47—H47	120.1
C8—C7—P1	121.1 (2)	C46—C47—C48	119.8 (3)
C12—C7—P1	120.0 (2)	C48—C47—H47	120.1
C7—C8—H8	120.1	C43—C48—H48	119.5
C7—C8—C9	119.8 (3)	C47—C48—C43	120.9 (3)
C9—C8—H8	120.1	C47—C48—H48	119.5
C8—C9—H9	119.7	C50—C49—Si1	120.3 (2)
C10—C9—C8	120.6 (3)	C54—C49—C50	116.9 (3)
C10—C9—H9	119.7	C54—C49—Si1	122.7 (2)
C9—C10—H10	119.9	C49—C50—H50	119.1
C11—C10—C9	120.2 (3)	C51—C50—C49	121.8 (3)
C11—C10—H10	119.9	C51—C50—H50	119.1
C10—C11—H11	120.2	C50—C51—H51	119.9
C10—C11—C12	119.6 (3)	C50—C51—C52	120.1 (3)
C12—C11—H11	120.2	C52—C51—H51	119.9
C7—C12—H12	119.6	C51—C52—H52	120.3
C11—C12—C7	120.9 (3)	C53—C52—C51	119.5 (3)
C11—C12—H12	119.6	C53—C52—H52	120.3
C14—C13—C18	119.1 (3)	C52—C53—H53	119.9
C14—C13—P1	122.2 (2)	C52—C53—C54	120.2 (3)
C18—C13—P1	118.7 (2)	C54—C53—H53	119.9
C13—C14—H14	119.8	C49—C54—C53	121.5 (3)
C13—C14—C15	120.3 (3)	C49—C54—H54	119.2
C15—C14—H14	119.8	C53—C54—H54	119.2
C14—C15—H15	119.9	H55A—C55—H55B	109.5
C16—C15—C14	120.3 (3)	H55A—C55—H55C	109.5
C16—C15—H15	119.9	H55B—C55—H55C	109.5
C15—C16—H16	119.9	Si1—C55—H55A	109.5
C15—C16—C17	120.2 (3)	Si1—C55—H55B	109.5
C17—C16—H16	119.9	Si1—C55—H55C	109.5
C16—C17—H17	120.2	C57—C56—Si1	122.1 (3)
C16—C17—C18	119.5 (3)	C61—C56—C57	116.4 (3)
C18—C17—H17	120.2	C61—C56—Si1	120.8 (3)
C13—C18—C17	120.5 (3)	C56—C57—H57	118.9
C13—C18—H18	119.7	C58—C57—C56	122.2 (4)
C17—C18—H18	119.7	C58—C57—H57	118.9
C20—C19—C24	119.2 (3)	C57—C58—H58	120.1
C20—C19—P2	122.2 (2)	C59—C58—C57	119.8 (4)
C24—C19—P2	118.6 (2)	C59—C58—H58	120.1
C19—C20—H20	119.8	C58—C59—H59	120.2
C21—C20—C19	120.5 (3)	C58—C59—C60	119.6 (3)
C21—C20—H20	119.8	C60—C59—H59	120.2
C20—C21—H21	119.9	C59—C60—H60	120.0
C20—C21—C22	120.2 (3)	C59—C60—C61	120.1 (4)
C22—C21—H21	119.9	C61—C60—H60	120.0
C21—C22—H22	120.2	C56—C61—H61	119.0
C23—C22—C21	119.6 (3)	C60—C61—C56	121.9 (4)
C23—C22—H22	120.2	C60—C61—H61	119.0
C22—C23—H23	119.8	O1—C62—Fe1	177.9 (3)
C22—C23—C24	120.3 (3)	O2—C63—Fe1	175.2 (3)
C24—C23—H23	119.8	O3—C64—Fe1	178.0 (3)
C19—C24—H24	119.9	C49—Si1—C55	107.20 (15)
C23—C24—C19	120.2 (3)	C49—Si1—C56	106.61 (14)
C23—C24—H24	119.9	C49—Si1—Fe1	110.49 (10)
C26—C25—C30	119.0 (3)	C55—Si1—C56	100.70 (15)
C26—C25—P2	124.7 (2)	C55—Si1—Fe1	114.30 (11)
C30—C25—P2	116.3 (2)	C56—Si1—Fe1	116.66 (11)
C25—C26—H26	119.7	C1—P1—C7	99.53 (13)
C25—C26—C27	120.6 (3)	C1—P1—Pt1	112.73 (10)
C27—C26—H26	119.7	C7—P1—Pt1	120.74 (10)
C26—C27—H27	119.9	C13—P1—C1	106.03 (14)
C28—C27—C26	120.2 (3)	C13—P1—C7	103.00 (14)
C28—C27—H27	119.9	C13—P1—Pt1	113.04 (10)
C27—C28—H28	120.0	C19—P2—Pt1	117.03 (10)
C27—C28—C29	120.0 (3)	C25—P2—C19	105.47 (13)
C29—C28—H28	120.0	C25—P2—C31	102.26 (13)
C28—C29—H29	119.8	C25—P2—Pt1	110.48 (9)
C28—C29—C30	120.4 (3)	C31—P2—C19	100.35 (13)
C30—C29—H29	119.8	C31—P2—Pt1	119.39 (9)
C25—C30—H30	120.1	C37—P3—Fe1	121.78 (10)
C29—C30—C25	119.9 (3)	C37—P3—Pt1	115.69 (10)
C29—C30—H30	120.1	C43—P3—C37	106.97 (14)
C32—C31—P2	122.8 (2)	C43—P3—Fe1	115.79 (9)
C36—C31—C32	118.2 (3)	C43—P3—Pt1	117.66 (10)
C36—C31—P2	118.9 (2)	Fe1—P3—Pt1	77.07 (3)
C31—C32—H32	119.9	C62—Fe1—Si1	78.43 (10)
C31—C32—C33	120.2 (3)	C62—Fe1—P3	88.17 (10)
C33—C32—H32	119.9	C62—Fe1—Pt1	93.70 (10)
C32—C33—H33	119.8	C63—Fe1—C62	98.17 (14)
C34—C33—C32	120.5 (3)	C63—Fe1—Si1	94.95 (10)
C34—C33—H33	119.8	C63—Fe1—P3	145.70 (10)
C33—C34—H34	120.0	C63—Fe1—Pt1	93.67 (10)
C33—C34—C35	120.1 (3)	C64—Fe1—C62	157.04 (15)
C35—C34—H34	120.0	C64—Fe1—C63	94.48 (15)
C34—C35—H35	120.2	C64—Fe1—Si1	81.46 (10)
C36—C35—C34	119.7 (3)	C64—Fe1—P3	92.12 (10)
C36—C35—H35	120.2	C64—Fe1—Pt1	104.57 (10)
C31—C36—H36	119.3	Si1—Fe1—Pt1	169.06 (3)
C35—C36—C31	121.4 (3)	P3—Fe1—Si1	119.32 (3)
C35—C36—H36	119.3	P3—Fe1—Pt1	52.16 (2)
C38—C37—P3	117.0 (2)	P1—Pt1—Fe1	102.64 (2)
C42—C37—C38	118.3 (3)	P2—Pt1—P1	101.80 (3)
C42—C37—P3	124.7 (2)	P2—Pt1—Fe1	154.80 (2)
C37—C38—H38	119.4	P3—Pt1—P1	153.40 (3)
C39—C38—C37	121.3 (3)	P3—Pt1—P2	104.70 (3)
C39—C38—H38	119.4	P3—Pt1—Fe1	50.77 (2)
C38—C39—H39	120.2	Cl1—C65—H65A	109.0
C38—C39—C40	119.5 (3)	Cl1—C65—H65B	109.0
C40—C39—H39	120.2	Cl2—C65—Cl1	112.9
C39—C40—H40	119.9	Cl2—C65—H65A	109.0
C41—C40—C39	120.3 (3)	Cl2—C65—H65B	109.0
C41—C40—H40	119.9	H65A—C65—H65B	107.8
			
C1—C2—C3—C4	−0.7 (5)	C36—C31—P2—C25	−166.4 (2)
C2—C1—C6—C5	2.1 (5)	C36—C31—P2—Pt1	71.4 (2)
C2—C1—P1—C7	104.5 (2)	C37—C38—C39—C40	0.0 (5)
C2—C1—P1—C13	−148.9 (2)	C38—C37—C42—C41	2.1 (4)
C2—C1—P1—Pt1	−24.7 (3)	C38—C37—P3—C43	171.3 (2)
C2—C3—C4—C5	1.0 (5)	C38—C37—P3—Fe1	−52.3 (3)
C3—C4—C5—C6	0.3 (6)	C38—C37—P3—Pt1	38.0 (3)
C4—C5—C6—C1	−1.9 (6)	C38—C39—C40—C41	1.3 (5)
C6—C1—C2—C3	−0.9 (5)	C39—C40—C41—C42	−0.9 (5)
C6—C1—P1—C7	−73.4 (3)	C40—C41—C42—C37	−0.8 (5)
C6—C1—P1—C13	33.2 (3)	C42—C37—C38—C39	−1.6 (5)
C6—C1—P1—Pt1	157.4 (2)	C42—C37—P3—C43	−8.4 (3)
C7—C8—C9—C10	0.4 (6)	C42—C37—P3—Fe1	128.0 (2)
C8—C7—C12—C11	0.9 (5)	C42—C37—P3—Pt1	−141.7 (2)
C8—C7—P1—C1	153.4 (3)	C43—C44—C45—C46	0.3 (5)
C8—C7—P1—C13	44.3 (3)	C44—C43—C48—C47	0.8 (4)
C8—C7—P1—Pt1	−82.9 (3)	C44—C43—P3—C37	−130.0 (2)
C8—C9—C10—C11	0.6 (6)	C44—C43—P3—Fe1	90.7 (2)
C9—C10—C11—C12	−0.8 (6)	C44—C43—P3—Pt1	2.2 (3)
C10—C11—C12—C7	0.1 (5)	C44—C45—C46—C47	0.2 (5)
C12—C7—C8—C9	−1.1 (5)	C45—C46—C47—C48	−0.2 (5)
C12—C7—P1—C1	−29.7 (3)	C46—C47—C48—C43	−0.4 (5)
C12—C7—P1—C13	−138.7 (3)	C48—C43—C44—C45	−0.8 (4)
C12—C7—P1—Pt1	94.1 (3)	C48—C43—P3—C37	60.0 (3)
C13—C14—C15—C16	1.1 (5)	C48—C43—P3—Fe1	−79.4 (3)
C14—C13—C18—C17	1.4 (5)	C48—C43—P3—Pt1	−167.8 (2)
C14—C13—P1—C1	−71.0 (3)	C49—C50—C51—C52	0.5 (5)
C14—C13—P1—C7	33.1 (3)	C50—C49—C54—C53	−0.5 (4)
C14—C13—P1—Pt1	165.0 (2)	C50—C49—Si1—C55	−170.7 (2)
C14—C15—C16—C17	0.5 (5)	C50—C49—Si1—C56	−63.6 (3)
C15—C16—C17—C18	−1.2 (5)	C50—C49—Si1—Fe1	64.1 (3)
C16—C17—C18—C13	0.2 (5)	C50—C51—C52—C53	−0.3 (5)
C18—C13—C14—C15	−2.1 (5)	C51—C52—C53—C54	−0.3 (5)
C18—C13—P1—C1	107.6 (3)	C52—C53—C54—C49	0.7 (5)
C18—C13—P1—C7	−148.3 (2)	C54—C49—C50—C51	−0.1 (4)
C18—C13—P1—Pt1	−16.3 (3)	C54—C49—Si1—C55	12.8 (3)
C19—C20—C21—C22	−0.6 (5)	C54—C49—Si1—C56	120.0 (3)
C20—C19—C24—C23	−3.0 (5)	C54—C49—Si1—Fe1	−112.3 (2)
C20—C19—P2—C25	64.3 (3)	C56—C57—C58—C59	−0.4 (6)
C20—C19—P2—C31	−41.7 (3)	C57—C56—C61—C60	1.3 (5)
C20—C19—P2—Pt1	−172.5 (2)	C57—C56—Si1—C49	168.1 (3)
C20—C21—C22—C23	−1.9 (5)	C57—C56—Si1—C55	−80.1 (3)
C21—C22—C23—C24	2.0 (5)	C57—C56—Si1—Fe1	44.2 (3)
C22—C23—C24—C19	0.5 (5)	C57—C58—C59—C60	0.9 (6)
C24—C19—C20—C21	3.1 (4)	C58—C59—C60—C61	−0.3 (6)
C24—C19—P2—C25	−114.5 (2)	C59—C60—C61—C56	−0.8 (6)
C24—C19—P2—C31	139.5 (2)	C61—C56—C57—C58	−0.7 (5)
C24—C19—P2—Pt1	8.8 (3)	C61—C56—Si1—C49	−22.2 (3)
C25—C26—C27—C28	1.4 (5)	C61—C56—Si1—C55	89.5 (3)
C26—C25—C30—C29	−0.1 (4)	C61—C56—Si1—Fe1	−146.2 (2)
C26—C25—P2—C19	−14.1 (3)	Si1—C49—C50—C51	−176.8 (2)
C26—C25—P2—C31	90.5 (3)	Si1—C49—C54—C53	176.1 (2)
C26—C25—P2—Pt1	−141.4 (2)	Si1—C56—C57—C58	169.4 (3)
C26—C27—C28—C29	−0.5 (5)	Si1—C56—C61—C60	−169.0 (3)
C27—C28—C29—C30	−0.8 (5)	P1—C1—C2—C3	−178.8 (2)
C28—C29—C30—C25	1.0 (5)	P1—C1—C6—C5	−180.0 (3)
C30—C25—C26—C27	−1.1 (4)	P1—C7—C8—C9	175.9 (3)
C30—C25—P2—C19	165.6 (2)	P1—C7—C12—C11	−176.1 (3)
C30—C25—P2—C31	−89.9 (2)	P1—C13—C14—C15	176.5 (2)
C30—C25—P2—Pt1	38.3 (2)	P1—C13—C18—C17	−177.2 (2)
C31—C32—C33—C34	−1.1 (5)	P2—C19—C20—C21	−175.7 (2)
C32—C31—C36—C35	−0.5 (4)	P2—C19—C24—C23	175.8 (2)
C32—C31—P2—C19	119.6 (3)	P2—C25—C26—C27	178.5 (2)
C32—C31—P2—C25	11.1 (3)	P2—C25—C30—C29	−179.8 (2)
C32—C31—P2—Pt1	−111.1 (2)	P2—C31—C32—C33	−176.5 (2)
C32—C33—C34—C35	0.5 (5)	P2—C31—C36—C35	177.2 (2)
C33—C34—C35—C36	0.0 (5)	P3—C37—C38—C39	178.6 (2)
C34—C35—C36—C31	−0.1 (5)	P3—C37—C42—C41	−178.3 (2)
C36—C31—C32—C33	1.0 (5)	P3—C43—C44—C45	−171.2 (2)
C36—C31—P2—C19	−57.9 (3)	P3—C43—C48—C47	171.1 (2)
